# A Class of Allopolyploidy Showing High Duplicate Retention and Continued Homoeologous Exchanges

**DOI:** 10.1093/gbe/evaf054

**Published:** 2025-03-19

**Authors:** Abbey Coppage, Esha Bhatnagar, Mitali Joshi, Mustafa Siddiqui, Logan McRae, Gavin C Conant

**Affiliations:** Department of Biological Sciences, North Carolina State University, Raleigh, NC, USA; Department of Biological Sciences, North Carolina State University, Raleigh, NC, USA; Department of Biological Sciences, North Carolina State University, Raleigh, NC, USA; Department of Biological Sciences, North Carolina State University, Raleigh, NC, USA; Department of Biological Sciences, North Carolina State University, Raleigh, NC, USA; Department of Biological Sciences, North Carolina State University, Raleigh, NC, USA; Genetics and Genomics Academy, North Carolina State University, Raleigh, NC, USA; Bioinformatics Research Center, North Carolina State University, Raleigh, NC, USA

**Keywords:** polyploidy, ohnolog retention, evolutionary model

## Abstract

We describe four ancient polyploidy events where the descendant taxa retain many more duplicated gene copies than has been seen in other paleopolyploidies of similar ages. Using POInT (the Polyploidy Orthology Inference Tool), we modeled the evolution of these four events, showing that they do not represent recent independent polyploidies despite the rarity of shared gene losses. We find that these events have elevated rates of interlocus gene conversion and that these gene conversion events are spatially clustered in the genomes. Regions of gene conversion also show very low synonymous divergence between the corresponding paralogous genes. We suggest that these genomes have experienced a delay in the return to a diploid state after their polyploidies. Under this hypothesis, homoeologous exchanges between the duplicated regions created by the polyploidy persist to this day, explaining the high rates of duplicate retention. Genomes with these characteristics arguably represent a new class of paleopolyploid taxa because they possess evolutionary patterns distinct from the more common and well-known paradigm of the rapid loss of many of the duplicated pairs created by polyploidy.

**Significance**

We describe four ancient polyploidies with unusually high levels of duplicate gene survival, as well as high levels of recent gene conversion. We argue that delayed rediploidization has retained and homogenized the duplicates in these genomes, in contrast to the more common pattern of rapid post-polyploidy duplicate gene loss.

## Introduction

Ancient polyploidy events have been found across the eukaryotic tree (Van de Peer et al. 2017; Hao et al. 2022) and are particularly abundant in the flowering plants and the ray-finned fishes. Although a newly formed tetraploid genome effectively contains four copies of every gene, the majority of the paleopolyploid genomes examined to date show very extensive *fractionation* (Thomas et al. 2006), meaning that most of the ohnologs (i.e. duplicate gene copies produced by polyploidy; Wolfe 2000) have been subsequently lost, due either to genetic drift or natural selection (Lynch and Conery 2000; Blanc and Wolfe 2004; De Smet et al. 2013). Many of these losses are likely to have occurred quite quickly relative to the timescale of speciation events, given the large number of shared ohnolog losses typically observed in the different genomes descending from the same polyploidy (Scannell et al. 2007; Hao et al. 2022).

Polyploidy events are commonly divided into allopolyploidies and autopolyploidies. Allopolyploidy occurs through the merging of genomes from two different, albeit related, species, meaning that it is in effect a genome doubling coupled to a hybridization. Autopolyploidy, on the other hand, occurs when the two genome copies derive from the same species (Stebbins 1947). Allopolyploidies appear to be more common among the surviving ancient polyploidy events (Barker et al. 2016; Hao et al. 2022). One piece of evidence for this claim is the fact that it is common to find that the ohnolog losses after polyploidy are unbalanced, with one of the progenitor genomes experiencing more losses than the other. This pattern is known as *biased* fractionation (Thomas et al. 2006; Woodhouse et al. 2010; Emery et al. 2018). Its presence is highly suggestive of the event in question being an allopolyploidy (Garsmeur et al. 2014), since it is difficult to understand how two initially identical genomes could produce loss biases (but see Makino and McLysaght 2012). The most often-invoked mechanism for biased fractionation is differential expression silencing of the subgenome with an excess of transposable elements. Such expression biases are then predicted to have the knock-on effect of favoring ohnolog loss from the suppressed genome (Freeling et al. 2012; Alger and Edger 2020). However, other mechanisms have recently been proposed: that the inherent kinetics of gene expression may give rise to expression and loss biases (An et al. 2024) or that the maternal subgenome may dominate in expression and gene preservation (Xu et al. 2023).

A prerequisite for any ohnolog losses after polyploidy is probably the suppression of homologous exchanges between the duplicated regions of the polyploid genome. Upon formation, at least for autopolyploids and allopolyploids of closely-related species, the similarities between the resulting homoeologous chromosomes will result in genetic exchanges between them at meiosis (Furlong and Holland 2002; Mandáková and Lysak 2018). This phase of polyploid evolution can be complex, with events such as entire chromosomes from one allopolyploid progenitor being replaced with the homoeologous copy from the other (Gaeta et al. 2007). Once such meiotic exchanges have ceased, we can refer to all or part of the polyploid genome as having become *diploidized*, such that each chromosome or chromosomal region undergoes recombination only with its sister chromosome (Robertson et al. 2017; Redmond et al. 2023) and not with the other progenitor subgenome.

**Fig. 1.**
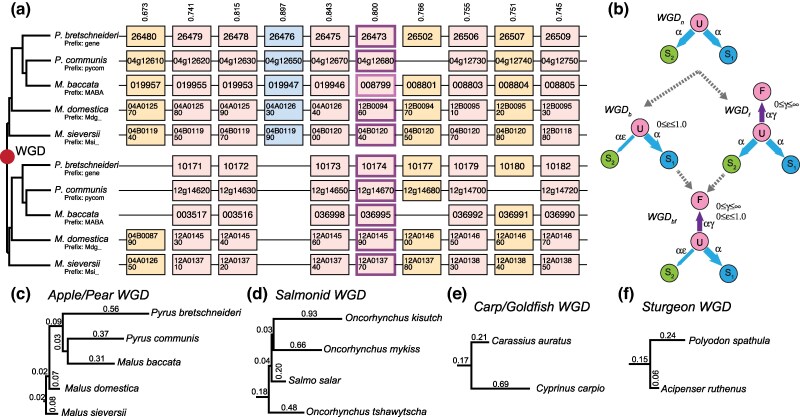
Modeling the evolution of four WGD events. a) An example region of ten ancestral genes that was duplicated in a WGD and is now found in two copies in five species of apples and pears. The upper and lower panels represent the two groups of orthologous regions (or *subgenomes*) formed by the WGD (abbreviated gene names are given in the boxes). This subgenome inference is more probable than the other 2^5^-1 possible subgenome assignments that we could make by swapping the paired regions from one or more species. The model's confidence *c* in the depicted subgenome assignments (as opposed to the other 2^5^-1 possible ones) is given by the numbers above the column (0 ≤ *c* ≤ 1). In the absence of homoeologous exchanges, fully retained ohnologs (columns without missing boxes; light pink) are expected to evolve under the mirrored species tree at left. A case of shared gene loss where all of the genes in the lower panel are missing is shown (blue), while other cases of individual losses have some duplicated and some single-copy genes (tan). b) The subgenome assignments in a) are made using the gene presence/absence data shown and evolutionary models of ohnolog loss. Four such nested loss models that differ with respect the presence of ohnolog pair fixation (rate γ) and biased fractionation (rate ε) are illustrated. Ohnolog pairs all start in state *U* (*U*ndifferentiated ohnolog) immediately post-WGD. Loss of the copy from subgenome 2 moves the pair to state *S*_1_ (only copy #1 retained) or symmetrically to *S*_2_. WGD_n_ is a null model without biased losses or duplicate fixation. The instantaneous ohnolog loss rate to both subgenomes is then equal (α). WGD_f_ allows for fixation of ohnologs which occur at a relative rate γ (0 ≤ γ < ∞): Ohnologs in *F* cannot undergo loss. WGD_b_ introduces biased losses through the ε (0 ≤ ε ≤ 1) parameter, making subgenome 1 increasingly favored as ε decreases. WGD_bf_ combines the WGD_b_ and WGD_f_ models. c to f) Inferred maximum likelihood topologies for the four events with the estimated branch lengths for each. Note that the branch lengths are not shown in the same scale across the panels.

In the absence of homoeologous exchanges, gene trees inferred from the ohnolog sequences from species sharing a polyploidy are expected to resolve to mirrored copies of the species tree (Fig. 1a). This pattern is seen because both ohnologs have experienced the same pattern of post-polyploidy speciation events. However, if speciation events occur prior to diploidization, any post-speciation recombination events will result in ohnologs that do not yield mirrored species trees (Robertson et al. 2017). On the basis of such conflicting phylogenetic patterns, authors have argued that the whole genome duplication (WGD) events in the salmonids and sturgeons, as well as the ancient teleost WGD, were followed by a prolonged period of homoeologous exchanges (Robertson et al. 2017; Parey et al. 2022; Redmond et al. 2023), meaning that diploidization was considerably delayed after these events. Indeed, in the case of the sturgeon WGD, prior work had argued for at least two independent genome duplications due to the conflicting gene trees (Crow et al. 2012; Cheng et al. 2021). However, a careful phylogenetic analysis resolved the corresponding gene trees as resulting from independent diploidization after a shared polyploidy in the lineages in question (Redmond et al. 2023).

The salmonid WGD is arguably the clearest case of “late diploidization” because extant species possessing it still show tetravalent pairing for some chromosomes (Phillips and Rab 2001; Braasch and Postlethwait 2012). Moreover, ohnologs found in these tetrasomically-inherited regions are less diverged from each other than are other ohnologs (Campbell et al. 2019). Since genetic exchanges between such homoeologous regions could also preserve ohnologs, such exchanges are also a plausible explanation for the excess of surviving ohnologs in the salmonids.

A clear open question is thus the frequency with which polyploidies with late diploidization are found in the tree of life. Using gene tree inferences to address this question is complicated by the fact that independent genome duplications can yield gene trees with topologies similar to those produced by late diploidization (Redmond et al. 2023). In this work, we sidestep the difficulties with using gene trees by using gene order, or *synteny*, data to detect late diploidization in polyploid genomes (Byrne and Wolfe 2005). We do so using our software package POInT (the Polyploidy Orthology Inference Tool; Conant and Wolfe 2008), which phylogenetically models the resolution of a polyploidy event using shared gene order and ohnolog losses (Fig. 1b). We have previously used POInT to analyze ten paleopolyploidy events, finding that neither the salmonid WGD nor the *Paramecium* octoploidy showed the extensive levels of ohnolog loss seen in the other eight “typical” polyploidy events (Hao et al. 2022). We also found elevated rate of interlocus gene conversion for both events. These conversion events “overwrite” (part of) the sequence of one ohnolog with the sequence from the other (Chen et al. 2007), possibly due to homoeologous recombination events. Because our analysis of the *Paramecium* genomes involved a phasing of a more recent and a more ancient tetraploidy (Hao et al. 2022), we were reluctant to draw overly firm conclusions from those observations at the time. Nonetheless, they are consistent with late diploidization for these two events and a more thorough exploration of its frequency is warranted.

Ancient genome duplications are known from the apples and pears, carp and goldfish, and the sturgeons (Van de Peer et al. 2017). Since our prior work, new genomes have become available from members of these three groups, allowing us to analyze them with POInT. Comparisons of the shared WGD in the carp and goldfish genomes have shown that some of the ohnolog pairs in these genomes are more similar to each other than they are to their orthologs, suggesting recent gene conversion (Li et al. 2021). There is also phylogenetic evidence for homoeologous exchanges between subgenomes in these lineages (Li et al. 2021) (note that despite these patterns, phylogenetics still supports a shared ancient event in these lineages; Xu et al. 2023). Our preliminary analyses, meanwhile, found elevated ohnolog survival rates across all three of these events. We therefore analyzed them, as well as the salmonid WGD, to assess whether they displayed hallmarks of late diploidization. We found that high ohnolog retention characterizing these four events reduces the phylogenetic loss signal available to POInT, but that sufficient signal remains for the robust detection of shared WGD events. We further found that all four events show spatial clustering in their retained ohnologs. They also show overly frequent and spatially clustered patterns of interlocus gene conversion, as well as very low synonymous divergence in these converted genes. We suggest that these four polyploidy events reflect a distinct class of paleopolyploid genome where diploidization is still incomplete even after tens or hundreds of millions of years of evolution.

## Results

### Modeling Post-Tetraploidy Ohnolog Losses Using Synteny Data and POInT

We inferred the regions of double-conserved synteny (DCS) created by ancient tetraploidy events in the salmonids, the apples and pears, carp and goldfish, and the sturgeons (Fig. 1a, Materials and Methods). For each event, we used POInT to infer the optimal phylogenetic topology from the DCS data using exhaustive tree search (Fig. 1c to f, Materials and Methods). Our datasets had 8,849, 10,513, 13,304, and 14,489 *pillars*, for the carp, apple/pear, sturgeon, and salmonid WGD events, respectively. Each pillar corresponds to an ancestral gene duplicated at the WGD and retained in at least one copy in every genome analyzed (Fig. 1a).

Using these optimal topologies, POInT allows the comparison of nested models of the losses of ohnolog copies along the phylogeny to test for both the presence of biased fractionation and of ohnolog fixation (Fig. 1b). All four events show bias toward one subgenome in the ohnolog losses (biased fractionation), consistent with these events having been allopolyploidies. For the apple/pear and salmonid events, there is also significant evidence for duplicate fixation (Table 1). However, once biased fractionation is accounted for, there is no evidence for fixation in the carp and sturgeon events, likely because only two genomes were studied in each case, too small a sample for the robust detection of fixation events.

**Table 1:** Models of post-WGD ohnolog loss

Event	WGD_n_ lnL^a^	WGD_b_ lnL^b^	*P* (WGD_n_ → WGD_b_)^c^	WGD_f_ lnL^d^	*P* (WGD_n_ → WGD_f_)^c^	WGD_bf_ lnL^e^	*P* (WGD_b_ → WGD_bf_)^c^	*P* (WGD_f_ → WGD_bf_)^c^
Salmon WGD	−42,326.95	−42,245.03	<10^−10^	−41,578.1	<10^−10^	−41,451.01^f^	<10^−10^	<10^−10^
Apple/pear WGD	−37,496.10	−36,979.41	<10^−10^	−37,482.16	1 × 10^−7^	−36,929.74^f^	<10^−10^	<10^−10^
Goldfish WGD	−17,609.54	−17,460.92^f^	<10^−10^	−17,609.52	<0.5	NA	NA	NA
Sturgeon WGD	−20,829.79^f^	−20,667.29^f^	<10^−10^	−20,802.28	<10^−10^	−20,666.71	0.28	<10^−10^

^a^ln-Likelihood of the observed ohnolog loss data under a model without either biased fractionation or duplicate fixation (null model; Fig. 1b). ^b^ln-Likelihood of the observed ohnolog loss data under the model allowing for biased fractionation but not duplicate fixation (Fig. 1b). ^c^*P*-value for the test of no improvement in fit from the first to second model: i.e. twice the difference in ln-likelihood compared to a chi-square distribution, 1 df in all cases. ^d^ln-Likelihood of the observed ohnolog loss data under the model allowing for ohnolog fixation but not biased fractionation (Fig. 1b). ^e^ln-Likelihood of the observed ohnolog loss data under the model allowing for both biased fractionation and ohnolog fixation (Fig. 1b). ^f^Optimal model.

### An Excess of Retained Ohnologs After Four WGD Events

In our previous work (Hao et al. 2022), the majority of the WGD events we studied retained relatively few of the ohnolog pairs created by the WGD, with percentages of ohnologs retained across all genomes ranging from about 12% (the yeast WGD) to 27% (At-α). In contrast, the events analyzed here retained 48% (carp WGD), 58% (salmonid WGD), 65% (sturgeon WGD), or even 70% (apple/pear WGD) of their ohnologs. The paramecium WGD also showed a high retention rate (49%) in our prior work: We do not analyze it here because we used an octoploid deconvolution approach when studying it that would be difficult to compare to these events (Hao et al. 2022).

### Confirmation of Single Polyploidy Events in Each of the Four Clades

One possible explanation for the apparent excess of ohnologs would be that we have mistaken several recent and independent WGD events for one ancient one, such that there has been less time for losses to occur than we assumed. We can test this hypothesis with POInT (Conant and Wolfe 2008). Using the goldfish and carp as an example, if these two species had experienced *independent* WGD events, there would be no true shared ohnolog losses between them, although by chance a few genes might have experienced parallel losses. In the context of POInT's models, such independent events could be modeled on a phylogenetic tree where the root branch is of zero length, corresponding to no shared losses.

We can therefore fit a model with a zero-length root branch to the DCS pillars and then use the resulting model parameters to *simulate* the evolution of a number of genomes under the assumption of independent polyploidies (Materials and Methods). If we fit to the resulting simulated polyploid genomes a model that allows the root branch to have a nonzero length, we can estimate a distribution of the apparent length of the root branch seen for cases where the true length was known to be zero (the apparent length will generally be nonzero because of parallel losses). If we compare that distribution to the length of the root branch for the real DCS data, we can compute a *P*-value for the test of the null hypothesis of a zero-length root branch having generated the observed root branch lengths in the real datasets.

**Fig. 2.**
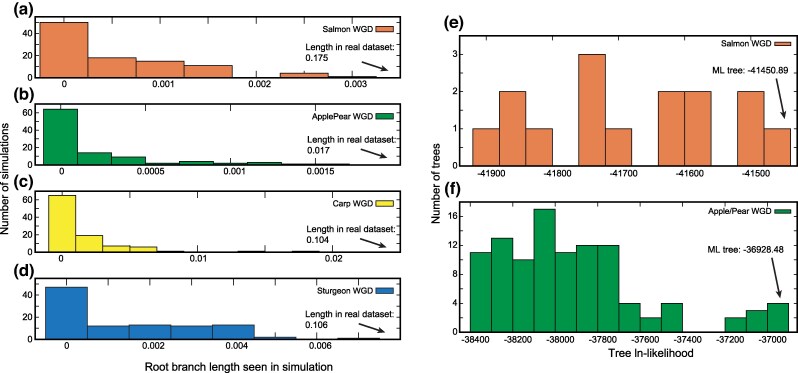
Four shared polyploidy events with phylogenetic signals of ohnolog loss. Panels a) to d) show 100 simulations of independent polyploidies fit to trees with the assumption of shared losses. In other words, the root branch is simulated as having zero length, corresponding to two independent polyploidies, but those simulated data are then fit with a model that allows the estimated length to be nonzero. Plotted on *x* is the estimated length of the root branch of the phylogeny for these simulations with a model allowing a nonzero root, showing the level of apparent shared ancestry expected for independent polyploidies. The arrows indicate the length of the root branch for the real data. Note that the WGD_n_ model was used for the sturgeon and goldfish/carp WGD events because the ε parameter in the WGD_b_ model can inflate the estimates of shared losses when only two genomes are analyzed (data not shown). Panels e) and f) illustrate the distribution of ln-likelihoods for all possible phylogenetic topologies for the salmonid and Apple/Pear WGD events, with the maximum likelihood tree indicated.

For all four events, the apparent lengths of the root branch for the simulated datasets are much smaller than that seen in the actual data (*P* < 0.01 in all cases; Fig. 2a to d), confirming that these are indeed shared ancient WGD events.

One could instead argue that, despite the speciation events observed, these polyploidies with high retention rates are so young that there was little time for ohnolog losses. However, their age is not noticeably younger than are other polyploidies with many more losses (Materials and Methods; supplementary figure, Supplementary Material online): In particular, the sturgeon event is the second oldest we have analyzed to date.

### The DCS Regions Contain Phylogenetic Signal Despite Yielding Unexpected Topologies

The topologies shown in Fig. 1c and d are not particularly similar to the expected relationships for the species in question (i.e. the members of the various genera are not monophyletic). One might therefore suspect that POInT's evolutionary models cannot recover any signal from these data and are hence inappropriate. However, we do not believe this to be the case. In Fig. 2e and f, we show the distribution of ln-likelihoods for all of the possible phylogenetic trees for the salmonids and apples and pears, respectively. In the absence of signal, we would expect all of the topologies to show roughly similar likelihoods: Instead, in both cases, there is a large group of topologies that show poor fit to the DCS data and a smaller number of topologies with higher likelihoods. Hence, while these datasets do not show ideal phylogenetic performance, there is still signal to be found.

### The Apple/Pear and Salmonid WGD Events Show Elevated and Spatially-Structured Patterns of Ohnolog Fixation

**Fig. 3.**
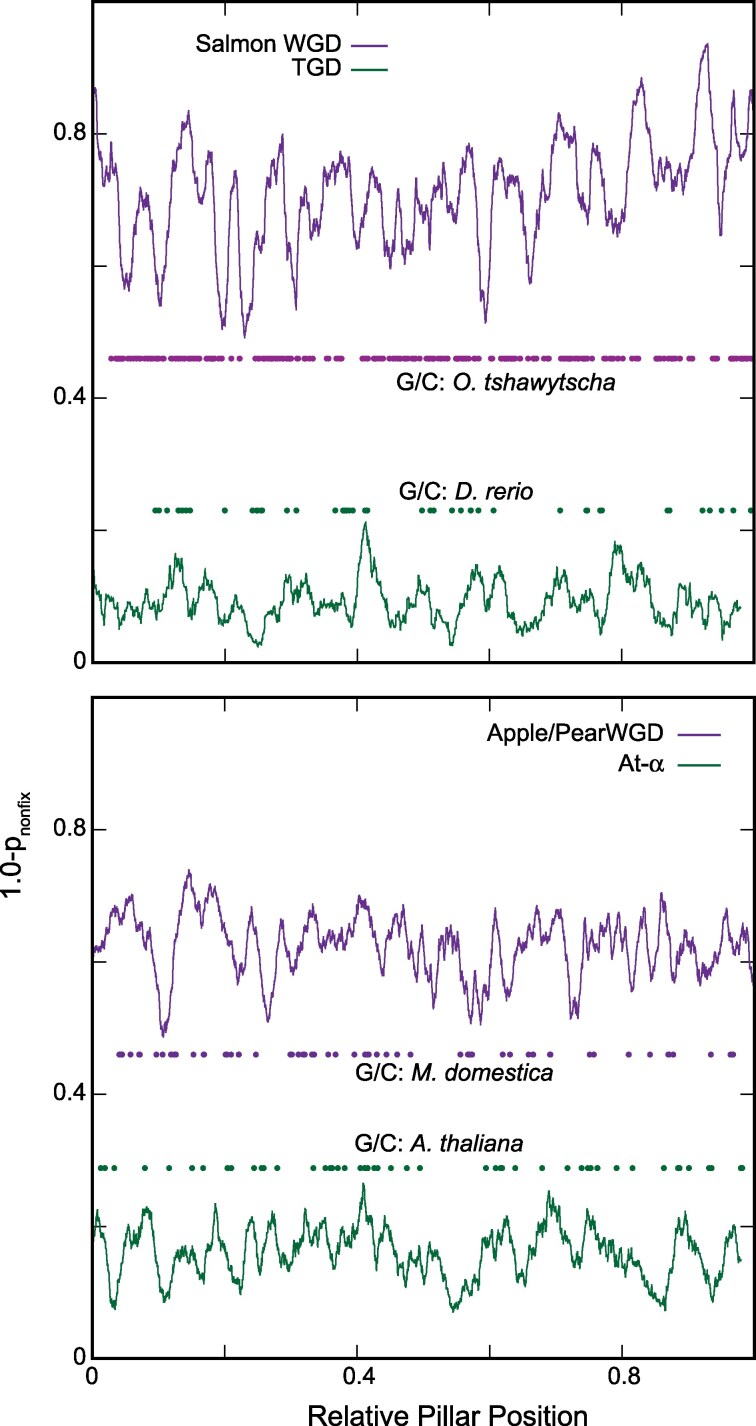
High and spatially-structured patterns of ohnolog fixation in the salmonid and apple/pear genomes. At each pillar, we computed the probability that *no* ohnolog pairs had been fixed (Materials and Methods). On the *y* axis is 1.0 minus that probability: in other words, the probability of at least one fixation at that pillar. The values shown are averaged across a sliding window of 2% of the size of the overall dataset (in terms of the number of pillars). Also shown are the position of all the significant gene conversion events for the genome with the most such events (dots colored by event; Materials and Methods and supplementary table S1, Supplementary Material online). On the *x* axis is the scaled pillar position (0 to 1) so that events with differing numbers of pillars can be compared. a) The salmonid WGD compared to the teleost genome duplication (TGD). b) The apple/pear WGD compared to the At-α WGD.

The WGD_bf_ model allows an ohnolog pair to become fixed along a branch of the phylogeny, such that that pair never undergoes loss later in the tree. For each pillar in the apple/pear and salmonid WGD events, we computed *p*_nonfix_: the probability that no such fixation had occurred along any branch for that pillar (Materials and Methods). If we compute 1.0 − *p*_nonfix_, we thus have the probability of at least one fixation event at that pillar. As shown in Fig. 3, both events are characterized by more duplicate fixation than other WGD events. A sliding window approach also suggests that these fixation rates are likely nonuniform across the genome, with hot and cold spots of fixation.

### These Polyploidy Events Show Elevated Rates of Recent Gene Conversion in the Coding Regions of Their Ohnologs

Using our previously described approach (Evangelisti and Conant 2010), we tested each surviving ohnologous gene pair with a clear ortholog for evidence of recent gene conversion. This test asks whether the two ohnolog sequences are more similar to each other than one is to its ortholog (Materials and Methods). Since the ohnologs had a common ancestor at the ancient WGD event while the orthologs diverged at a more recent speciation event, such a pattern is evidence of gene conversion since that speciation.

These genomes generally show a higher relative frequency of gene conversion than is observed in comparisons for the teleost genome duplication (TGD) or the recent WGD in *Arabidopsis thaliana* and its relatives. Moreover, unlike those events, the gene conversions in these genomes show spatial clustering (supplementary table S1, Supplementary Material online).

**Fig. 4.**
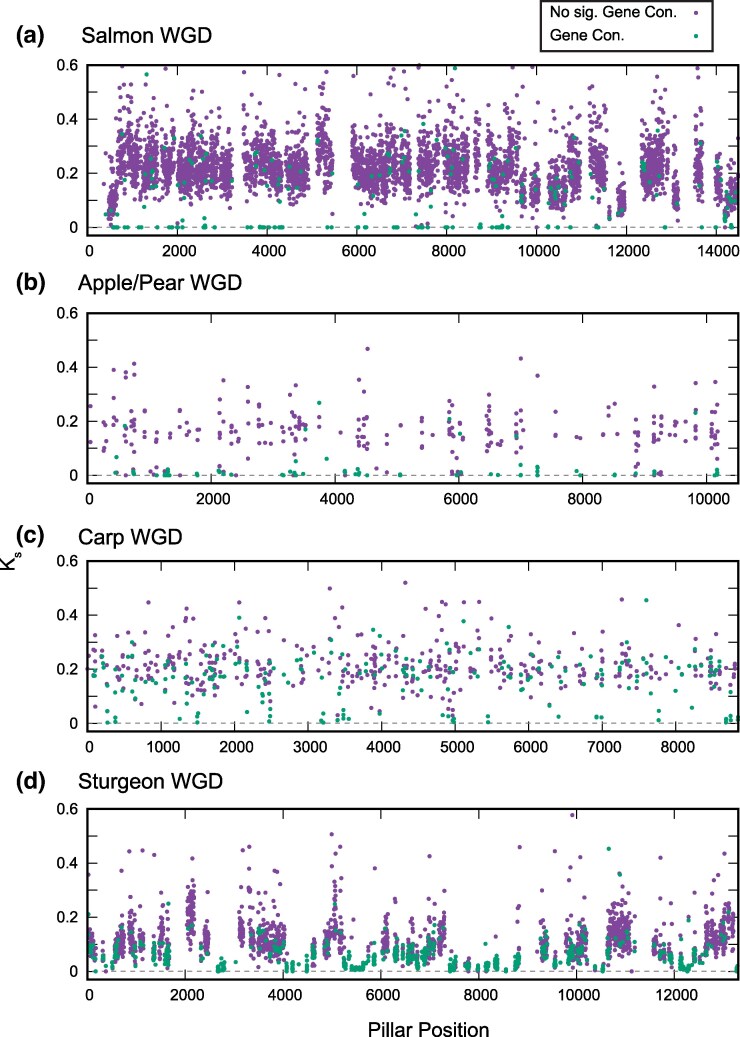
Low synonymous divergence between paralogs with evidence of recent gene conversion. For each of the four WGD events, we selected the genome with the largest number of cases of gene conversion (supplementary table S1, Supplementary Material online). We then computed the pairwise synonymous divergence (*K*_s_) for all paralogs used in the gene conversion analysis and plotted these values for paralog pairs with (*P* < 0.05, supplementary table S1, Supplementary Material online; green) and without (*P* > 0.05, supplementary table S1, Supplementary Material online; purple) evidence of recent gene conversion. *K*_s_ often approaches 0 (dashed line) for pairs with evidence for conversion. The *x* axis represents the relative position of the ohnolog pair in our ancestral genome order reconstruction. Cases where *K*_s_ > 0.6 are omitted for clarity. a to d) Plots for the four WGD events considered.

To get a sense of whether these gene conversion events are recent, we compared the synonymous divergence of the paralogs (*K*_s_, number of synonymous substitutions per synonymous site) between pairs with evidence of gene conversion to those without. As shown in Fig. 4, many of the paralog pairs with evidence for recent gene conversion show synonymous divergence approaching 0, consistent with an ongoing process of gene conversions in these taxa.

## Discussion

In most new-formed polyploids, recombinations and genetic exchanges between homoeologous chromosomes are extensive (Gaeta et al. 2007). Exceptions include the hexaploid common wheat, where the *Ph1* gene suppresses such recombinations, giving rise to “diploid-like” meiotic pairing (Riley and Chapman 1958; Zhang et al. 2020). Among the much older paleopolyploidies, homoeologous exchanges appear to have largely ceased, as recent gene conversion in these taxa is markedly less frequent and most of the duplicated genes have commonly been lost (Casola, Conant, and Hahn 2012; Hao et al. 2022; Yang et al. 2023). These facts suggest that such genomes long ago experienced full diploidization.

**Fig. 5.**
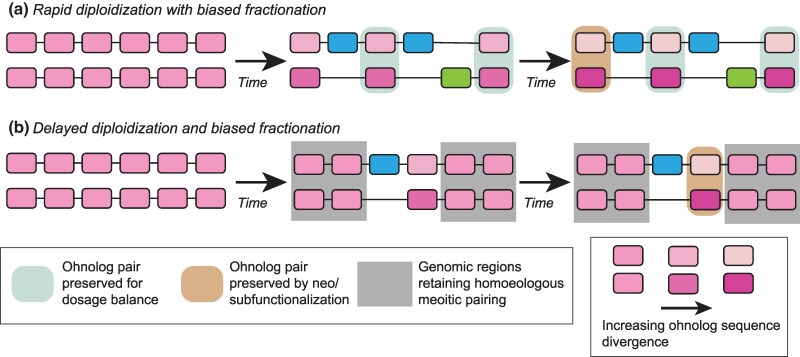
Two models for post-polyploidy genome evolution. a) In a “typical” tetraploidy, many or most ohnolog pairs are reduced to single-copy by genetic drift fairly quickly after the event. Ohnologs may be protected from loss by a need for dosage balance (teal) or, over longer timescales, by functional divergence (tan). One of the two progenitor subgenomes may be favored in the losses (blue verses green, i.e. biased fractionation). b) For the polyploidies considered here, some regions of the genome (gray) maintain meiotic pairing between the progenitor subgenomes. As a result, ohnolog pairs in these regions are maintained and experience limited sequence divergence (observable in our data as gene conversions). Other regions of the genome may follow patterns more similar to standard tetraploidies. The difference in shading between ohnolog pairs illustrates their sequence divergence (or lack thereof).

Here, we describe four paleopolyploidy events where the descendent genomes have three unusual features: (i) They have high rates of ohnolog retention (supplementary figure, Supplementary Material online), (ii) those retentions are spatially structured along chromosomes (Fig. 3), and (iii) they exhibit high rates of gene conversion (supplementary table S1, Supplementary Material online). We propose that a process of spatially heterogeneous and incomplete diploidization after these polyploidies can explain all three of these observations, justifying our claim of these events representing a new class of paleopolyploid genome. Figure 5 presents a cartoon view of these two models of post-polyploidy evolution.

In support of this hypothesis, we note that such continued tetrasomic pairing is already known for salmonids (Phillips and Rab 2001; Braasch and Postlethwait 2012), where some regions of the chromosomes undergo such pairing and others do not. When such pairings occur, they will tend to locally both preserve ohnolog pairs and homogenize their sequences, accounting for the high rates of ohnolog retention and gene conversion among the salmonids. We also note that in synthetic wheat tetraploids where homoeologous recombination does occur, it tends to be confined to gene bodies, in contrast to homologous pairing that is concentrated in other chromosomal regions (Zhang et al. 2020), an interesting parallel to the gene conversion events seen here. Furthermore, looking at Fig. 4a and d, we see suggestions of local regions of lower and higher *K*_s_, which could be explained by a more recent or more ancient loss of tetrasomic pairing. This observation would be consistent with the spatial structuring seen in the gene conversions and ohnolog fixations. We therefore propose that these genomes preserve in their gene loss and conversion patterns a history of slow rediploidization. How slow? One of our more striking results is that, for all four events, we find regions marked by gene conversion that also include ohnolog pairs with little to no synonymous or nonsynonymous divergence between them (supplementary table S2, Supplementary Material online). We are only able to explain this pattern on the hypothesis that diploidization is in fact still ongoing in these lineages, with gene conversion events between the ohnologs continuing essentially until the present.

The evolution of “conventional” paleopolyploid genomes appears to be governed by the degenerative effects of genetic drift eliminating ohnologs on the one hand (Li 1980; Lynch and Conery 2000) and preservation of those ohnologs to maintain dosage balance (Freeling 2009; Birchler and Veitia 2012) on the other (see cartoon in Fig. 5). This second force maintains ohnolog pairs because the loss of one copy can change the corresponding protein's expression level relative to other gene products that it might physically interact with, regulate, be regulated by or otherwise functionally associate with (Veitia et al. 2013; Pires and Conant 2016). That such relative dosage imbalances can be detrimental is shown by the correlation between a human copy-number variant's propensity to be pathogenic and the tendency of the gene containing it to have maintained its copy number status over evolutionary time (Rice and McLysaght 2017). The new class of paleopolyploidy just described is presumably still subject to the constraints of dosage balance. It appears, however, that the propensity of drift to remove duplicated material is now opposed by recombination with homoeologous chromosomes (Fig. 5).

From a methodological perspective, these patterns will have the unfortunate consequence of confounding phylogenetic inferences made using either the ohnolog losses (as for POInT) or the sequences themselves. In the case of POInT, the low levels of ohnolog loss yield little information for topology inference, and indeed, the topologies in Fig. 1 are not particularly sensible. At least among the surviving ohnologs, traditional phylogenetic methods will also tend to be misled due to the overwriting of the phylogenetic signal by gene conversion. We do note, however, that there are now sequence evolution models available that include gene conversions (Ji et al. 2016; Yang et al. 2023).

It is also worth speculating about the larger consequences of this new type of polyploidy. It has long been recognized that extending the “half-life” of a duplicated gene pair gives a relatively greater chance for functional innovations to occur in those duplicates (Rastogi and Liberles 2005; He and Zhang 2006). Of course, gene conversions can limit functional divergence since the duplicates maintain very similar sequences. However, this limitation is not absolute, as, in yeast, there are duplicates with amino acid sequences that are kept identical by gene conversion that are nonetheless functionally distinct due to differing expression patterns (Ni and Snyder 2001; Komili et al. 2007). Whether these patterns of retention and conversion will persist indefinitely is also unclear, although the salmonid genome duplication is believed to be many tens of millions of years old (Braasch and Postlethwait 2012), while the sturgeon event is even older than this (Redmond et al. 2023). Polyploidy events continue to surprise us with their complexities, and there is no reason to believe that more surprises do not await us as new genomes allow us to consider new events.

## Materials and Methods

In addition to the salmonid WGD already described (Hao et al. 2022), we analyzed shared WGD events in the apples and pears (Van de Peer et al. 2017), sturgeons (Redmond et al. 2023), and the event shared between common carp and goldfish (Chen et al. 2019b) using POInT (Conant and Wolfe 2008; Emery et al. 2018).

### Genomes

We included five genomes in our analysis of apples and pears: those of *Malus domestica* (domestic apple; Daccord et al. 2017), *Malus baccata* (Chinese wild apple; Chen et al. 2019a), *Malus sieversii* (central Asian wild apple; Sun et al. 2020), *Pyrus bretschneideri* (Chinese white pear; Xue et al. 2018), and *Pyrus communis* (European pear; Chagné et al. 2014 ). The almond genome (*Pyrus dulcis*) was used as the nonpolyploid outgroup reference (Alioto et al. 2020). These six genomes were downloaded from the Genome Database for Rosaceae (Jung et al. 2019). For the carp/goldfish event, we used the genomes of *Cyprinus carpio* (common carp; Xu et al. 2014) and *Carassius auratus* (goldfish; Chen et al. 2019b). The genome of *Cyprinodon variegatus* (sheepshead minnow; Lencer et al. 2017) was used as a non-tetraploid genome reference. All three genome sequences were obtained from Ensembl release 107 (Cunningham et al. 2022). For the sturgeon/paddlefish event, we compared the genomes of *Acipenser ruthenus* (sterlet sturgeon; Du et al. 2020) and *Polyodon spathula* (paddlefish; Cheng et al. 2021) to the genome of the outgroup *Lepisosteus oculatus* (spotted gar; Braasch et al. 2016). The sturgeon and paddlefish genomes were both obtained from NCBI's GenBank (Sayers et al. 2022); the gar genome was taken from Ensembl release 98.

### Inference of Blocks of Shared DCS Produced by Paleotetraploidies

For all three polyploidy events above, we used a three-step method to identify the blocks of DCS that survive from the ancient tetraploidies. The first step is a homology search of each of the nine tetraploid genomes against the unduplicated outgroup (i.e. *P. dulcis*, *C. variegatus*, or *L. oculatus*). This search uses GenomeHistory (v. 2.0; Conant and Wagner 2002) and BLASTP (v. 2.7.1; Altschul et al. 1997) to identify homologous pairs of genes from the outgroup and tetraploid genomes: these pairs are then filtered, aligned, and their synonymous and nonsynonymous divergence computed (Li 1997). For the comparison of apple and pear genomes to almond, we required a BLAST *E* ≤ 10^−10^ and 70% or greater amino acid identity to retain a homolog pair. The corresponding figures for the carp/goldfish and the sturgeon events were *E* ≤ 10^−8^ and 60% or greater identity.

The second step of the analysis was a search for DCS blocks between each tetraploid genome and its respective outgroup (Hao and Conant 2022). This search uses simulated annealing to maximize the size of the inferred DCS blocks. Successively longer runs of these searches were made until this DCS score converged.

The third and final step was to merge the five sets of DCS blocks inferred from the apple and pear genomes and the two sets of DCS blocks from the carp/goldfish and sturgeon/paddlefish into a single set of blocks each. We then optimized the order of the homoeologous pillars within these three merged sets of DCS blocks, again using simulated annealing. These ordered pillars could then be analyzed with POInT (Hao and Conant 2022).

### Modeling of Ohnologous Gene Losses With POInT

For all four WGDs, we modeled the loss of ohnologous genes using four related models of ohnolog loss (Fig. 1b). For each model, we use POInT (v. 1.61) to search for the maximum likelihood phylogenetic topology using an exhaustive tree search. In other words, we sought the topology of highest likelihood for the five apple/pear species from among the 115 possible rooted topologies and similarly for the 15 possible topologies for the four salmonid species. The topologies of the goldfish and carp and of the sturgeons and paddlefish are trivial. We computed the significance of the improvement in model fit for more complex models using a likelihood ratio test (Sokal and Rohlf 1995). These inferences and DCS blocks can be visualized at the POInT_browse_ portal (wgd.statgen.ncsu.edu; Siddiqui and Conant 2023).

### Dating the Four Polyploidy Events Considered

Dating polyploidy events can be contentious. As a rough approximation, we used the TimeTree package (Kumar et al. 2017) to compare the earliest estimated divergence date between any of the pairs of species in each of our four datasets. We compared these divergence values to those from eight other polyploidies we had previously analyzed (Hao et al. 2022; McRae et al. 2022). We then computed the minimum, maximum, and average proportion of homoeologs that retained both/all copies of the genes in question for all of these events (supplementary figure, Supplementary Material online). We note that the date for the salmonid WGD in particular is likely an underestimate, as the whitefishes and graylings are thought to share this event (Phillips and Rab 2001; Braasch and Postlethwait 2012): including them in the TimeTree comparisons pushes the date of this event back to roughly 55 MYA.

### Test for a Single, Shared Polyploidy Event

To test whether the four WGD events considered might represent two independent WGD events in each lineage, we fit a model to each dataset that forced the length of the root branch of the optimal topology to have zero length. This restriction corresponds to the assumption of no shared gene losses between the lineages separated by the first speciation event in the tree. We then simulated 100 sets of genomes from this topology and model parameters. To each simulated dataset, we fit a model with a zero-length root branch (Model 1) and a model where the root branch was allowed to have an arbitrary length (Model 2). We then computed the difference in ln-likelihood between these two models (Conant and Wolfe 2008). We also compared the resulting distribution of the length of the root branch in the simulations analyzed under Model 2 to the observed length of the root branch in the actual datasets (Fig. 2a). Using either the distribution of differences in log-likelihood between Model 2 and Model 1 or the distribution of estimated root branch length from the simulations resulted in the same rejection of the null hypothesis of no improvement in fit for the real data when allowing a nonzero length root (Fig. 2).

### Analysis of Fixation Frequency Across the Genome

To assess whether the patterns of ohnolog fixation were uniform throughout the genome, we adopted a sliding window measure of fixation frequency. At each pillar, we computed *p*_fix_*^i^*: the probability that the ohnolog gene pair at that pillar was fixed for taxa *i*, using the state conditional probabilities for that pillar (Emery et al. 2018). We then computed *p*_nonfix_: the probability of *no* fixation events across that pillar as


(1) $$p_{nonfix}=\prod_{i}\left(1-p_{\mathrm{fix}}^{i}\right).\;$$

In other words, *p_nonfix_* gives the probability of no fixation events across any genomes at that pillar.

### Tests for Gene Conversion

We tested ohnologous gene pairs for evidence of recent gene conversion events with an approach similar to our previous work (Evangelisti and Conant 2010). For a given genome with a WGD, we first identified pairs of ohnologous genes *G*_1_ and *G*_2_ for which we could identify the syntenic ortholog *O* of *G*_1_ in a close polyploid relative with high confidence *c*. The value of *c* is taken from the orthology confidence estimates in POInT; we used a value of *c* ≥ 0.9 for the TGD, At-α, salmonid, and sturgeon events and *c* ≥ 0.8 for the carp and apple/pear WGD events. We used lower cutoffs for the latter two events because they demonstrated low orthology confidence values overall. Results from using a cutoff of *c* ≥ 0.9 for the apple/pear events were qualitatively similar, but three of the five comparisons did not show significant clustering because of the small number of ohnologs tested. As an aside, we note that the low orthology confidences are due to the small number of shared gene losses for these events: that small number of losses is in turn due to the high ohnolog retention rates discussed here.

We aligned the translated coding sequences of these three genes (*G*_1_, *G*_2_, and *O*) with T-Coffee v. 13.45 (Notredame et al. 2000) and deduced the corresponding nucleotide alignment. We then fit the Muse and Gaut/Goldman and Yang model of codon evolution (Goldman and Yang 1994; Muse and Gaut 1994) to the sequence triplet as previously described (Conant and Wagner 2003). The result of that analysis is a branch-specific value of *K*_a_ (number of nonsynonymous substitutions per nonsynonymous site) for each of the three genes. The signal of gene conversion for such a triplet is when *K*_a_ for *O* ($K_{a}^{O}$) is greater than *K*_a_ for both *G*_1_ and *G*_2_ ($K_{a}^{G_{1}}$ and $K_{a}^{G_{2}}$). Recall that *G*_1_ and *O* last shared a common ancestor at their (recent) speciation event, while *G*_1_ and *G*_2_ last shared a common ancestor at the (more ancient) WGD event. Hence, in the absence of gene conversion, $K_{a}^{O}$ should be less than $K_{a}^{G_{2}}$. When $K_{a}^{O}>K_{a}^{G_{1}}$, $K_{a}^{G_{2}}$, we fit an alternative model that forced $K_{a}^{O}=K_{a}^{G_{2}}$. We computed the significance of the inferred gene conversion by comparing the log-likelihoods of the original model and the constrained model where $K_{a}^{O}=K_{a}^{G_{2}}$ using a likelihood ratio test with 1 df (Sokal and Rohlf 1995).

Among the pillars with significant evidence for a gene conversion event (*P* ≤ 0.05), we tested for spatially clustering as follows. For each such event, we computed the distance to the closest other pillar with a significant gene conversion event and took the average of this value across all significant events. We then conducted 1,000 randomizations where we laid the same number of events uniformly on the pillars and computed the same distance statistic. We then computed the proportion of the simulations with a smaller mean distance between gene conversion events than the observed distance for the real gene conversions (supplementary table S1, Supplementary Material online). For all ohnolog pairs in the gene conversion analysis, we also computed their pairwise synonymous divergence (*K*_s_) again using the codon model of Muse and Gaut/Goldman and Yang (Goldman and Yang 1994; Muse and Gaut 1994). The list of all significant cases of gene conversion is available from POInT_browse_ (https://wgd.statgen.ncsu.edu/Downloads/HighDupl_Poly_sig_GC_list.txt).

## Supplementary Material

evaf054_Supplementary_Data

## Data Availability

DCS blocks, coding region sequences, optimal phylogenetic trees, and loss models are available from POInT_browse_ (https://wgd.statgen.ncsu.edu). A list of all identified, significant cases of gene conversion for the 13 genomes considered here is also available from POInT_browse_ (https://wgd.statgen.ncsu.edu/Downloads/HighDupl_Poly_sig_GC_list.txt). POInT (v. 1.61) is available from GitHub (https://github.com/gconant0/POInT). Software for the analysis of sequence triplets is also available at GitHub (https://github.com/gconant0/like_tri_test).
