# Supplementary material for: A Class of Allopolyploidy Showing High Duplicate Retention and Continued Homoeologous Exchanges
Source: Genome Biol Evol. 2025 Mar 19;17(4):evaf054. doi: 10.1093/gbe/evaf054 (PMC11965797; doi:10.1093/gbe/evaf054)
Supplement: evaf054_Supplementary_Data [file evaf054_supplementary_data.zip › SupplementalTables.docx]

**Supplemental Table 1**

| **Event** | **Focal Genome^a^** | **Outgroup Genome^b^** | **#Gene Con. Events^c^** | **Prop. of**  **Homoeologs tested^d^** | **Mean dist. to nearest GC^e^** | **Avg. mean dist .(random)^f^** | ***P^g^*** |
| --- | --- | --- | --- | --- | --- | --- | --- |
| Apple/Pear^h^ | *M. baccata* | *P. communis* | 41 | **0.091** | 61.1 | 124.8 | **<0.001** |
|  | *M. domestica* | *M. baccata* | 115 | **0.195** | 20.1 | 45.4 | **<0.001** |
|  | *M. sieversii* | *M. domestica* | 114 | **0.167** | 19.1 | 45.8 | **<0.001** |
|  | *P. bretschneideri* | *M. baccata* | 43 | **0.124** | 55.9 | 118.9 | **<0.001** |
|  | *P. communis* | *M. baccata* | 22 | **0.049** | 146.4 | 229.5 | **0.025** |
| Salmonid WGD^i^ | *O. kisutch* | *S. salar* | 233 | **0.024** | 22.2 | 31.2 | **<0.001** |
|  | *O. mykiss* | *S. salar* | 225 | **0.024** | 25.6 | 32.4 | **<0.001** |
|  | *O. tshawytscha* | *S. salar* | 716 | **0.074** | 5.8 | 10.4 | **<0.001** |
|  | *S. salar* | *O. kisutch* | 359 | **0.035** | 13.1 | 20.3 | **<0.001** |
| Carp WGD^h^ | *C. auratus* | *C. carpio* | 299 | **0.325** | 8.8 | 15.1 | **<0.001** |
|  | *C. carpio* | *C. auratus* | 48 | **0.077** | 59.6 | 90.6 | **0.002** |
| Sturgeon WGD^i^ | *A. ruthenus* | *P. spathula* | 1109 | **0.230** | 2.38 | 6.26 | **<0.001** |
|  | *P. spathula* | *A. ruthenus* | 271 | **0.062** | 12.7 | 24.8 | **<0.001** |
| At-α^i^ | *A. arabicum* | *A. thaliana* | 0 | 0 | NA | NA | NA |
|  | *A. lyrata* | *A. thaliana* | 0 | 0 | NA | NA | NA |
|  | *A. thaliana* | *A. lyrata* | 56 | 0.019 | 62.2 | 63.8 | >0.1 |
|  | *C. rubella* | *A. thaliana* | 0 | 0 | NA | NA | NA |
|  | *E. salsugineum* | *S. parvula* | 53 | 0.18 | 72.9 | 67.1 | >0.1 |
|  | *S. parvula* | *E. salsugineum* | 68 | **0.024** | 49.1 | 52.7 | >0.1 |
| TGD^i^ | *A. mexicanus* | *D. rerio* | 8 | 0.011 | 224.1 | 307.0 | >0.1 |
|  | *D. rerio* | *A. mexicanus* | 48 | 0.061 | 42.2 | 57.4 | **0.02** |
|  | *G. aculeatus* | *O. niloticus* | 4 | 0.005 | 1108.5 | 571.9 | >0.1 |
|  | *O. niloticus* | *O. latipes* | 24 | **0.029** | 108.6 | 112.1 | >0.1 |
|  | *O. latipes* | *X. maculatus* | 8 | 0.011 | 390.6 | 315.3 | >0.1 |
|  | *T. rubripes* | *T. nigroviridis* | 7 | 0.009 | 469.9 | 349.2 | >0.1 |
|  | *T. nigroviridis* | *T. rubripes* | 9 | 0.012 | 336.1 | 284.7 | >0.1 |
|  | *X. maculatus* | *O. latipes* | 14 | 0.019 | 201.1 | 182.5 | >0.1 |

a: Genome in which homoeolog pairs were tested for smaller K_a_ values than was seen to the ortholog of one (*Methods*).

b: Genome from which the orthologous gene was taken for the gene conversion test (*Methods*).

c: The number of homoeologous pairs from the focal genome for which K_a_ for both members of the pair was significantly smaller than the K_a_ value for the orthologous gene (*P≤*0.05, likelihood-ratio test, *Methods*)

d: Proportion of all homoeologous pairs tested for which the evidence of gene conversion was significant. Values in bold are greater than an (arbitrary) threshold of 0.02.

e: Across all significant instances of gene conversion, the average of the distance from a significant pair to another significant pair, where distance is measured in POInT pillar numbers (*Methods*).

f: Across 1000 random simulations of a uniformly distributed set of gene conversion events, the average of the minimum distance between pairs of gene conversion events (*c.f.,* the prior column).

g: *P-*value for the test of a smaller average distance between pairs of gene conversion events seen in the real data compared to uniformly distributed gene conversions. Bold text are cases significant at *P≤*0.05.

h: A POInT orthology confidence value of 0.8 was used for these events because of the low overall orthology confidence from the POInT analyses in these events.

i: A POInT orthology confidence value of 0.9 was used for these events.

**Supplemental Table 2**

| **Event** | **Focal Genome^a^** | **Orthology confidence cutoff^b^** | **#Pillars tested^c^** | **K_s_<10^-4^, K_a_<10^-4 d^** | **K_s_<0.01^e^** |
| --- | --- | --- | --- | --- | --- |
| Apple/Pear^h^ | *M. domestica* | 0.8 | 358 | 16 | 67 |
| Salmonid WGD^i^ | *O. tshawytscha* | 0.9 | 5129 | 337 | 357 |
| Carp WGD^h^ | *C. auratus* | 0.4 | 670 | 0 | 8 |
| Sturgeon WGD^i^ | *A. ruthenus* | 0.9 | 2724 | 13 | 88 |

a: Polyploid genome with the largest number of significant gene conversion events (*Methods*).

b: Confidence cutoff for the identification of homoeologous regions (*Methods*).

c: Number of pillars with confidence greater than the cutoff in the previous column

d: Number of homoeologous genes with both K_s_ and K_a_ having values less than 10^-4^

d: Number of homoeologous genes with K_s_ less than 0.01. The cases in the previous column are included in this count.
