# Supplementary material for: A Class of Allopolyploidy Showing High Duplicate Retention and Continued Homoeologous Exchanges
Source: Genome Biol Evol. 2025 Mar 19;17(4):evaf054. doi: 10.1093/gbe/evaf054 (PMC11965797; doi:10.1093/gbe/evaf054)
Supplement: evaf054_Supplementary_Data [file evaf054_supplementary_data.zip › Supplemental_Figures.pdf]

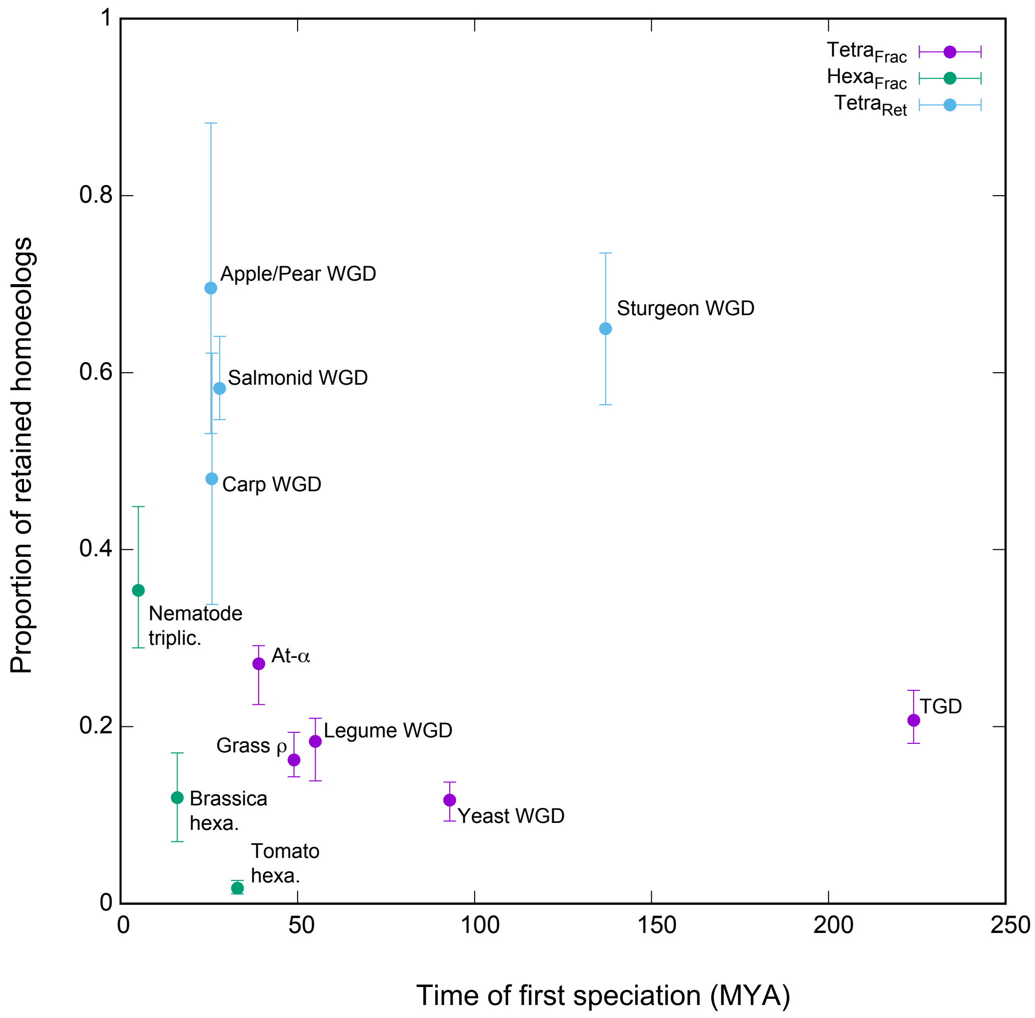

Supplemental Figure: Tetraploidies with high duplicate retention are not recently formed. We compare the minimum age (*x*-axis) and the proportion of retained homoeologs (*y*-axis) for the four tetraploidies considered here (blue) to a selection of tetraploidy (purple) and hexaploidy (green) events we have previously analyzed (*Methods*). To estimate the minimum age of the event, we found the oldest dated split between the genomes analyzed using the TimeTree package (*Methods*). We then compared that value to the minimum and maximum (bars) and average (points) proportion of homoeolog pairs retained from that event across the genomes studied. Notice that particularly for the salmonid WGD, the date shown probably under-estimate the polyploidy's age, as the graylings and whitefishes that share this event are not included among our genome sample (*Methods*).
